# Intelligent Neurovascular Imaging Engine (INIE): Topology-Aware Compressed Sensing and Multimodal Super-Resolution for Real-Time Guidance in Clinically Relevant Porcine Stroke Recanalization

**DOI:** 10.3390/diagnostics16040615

**Published:** 2026-02-20

**Authors:** Krzysztof Malczewski, Ryszard Kozera, Zdzislaw Gajewski, Maria Sady

**Affiliations:** 1 Institute of Information Technology, Warsaw University of Life Sciences, Nowoursynowska St. 159, Building 34, 02-776 Warsaw, Poland; ryszard_kozera@sggw.edu.pl; 2 Center of Translational Medicine, Warsaw University of Life Sciences, Nowoursynowska St. 100, 02-797 Warsaw, Poland; maria_sady@sggw.edu.pl

**Keywords:** neurovascular imaging, adaptive acquisition, topology-preserving reconstruction, super resolution, compressed sensing, sensor-informed imaging, uncertainty quantification, MR–PET, stroke imaging, clinical decision support

## Abstract

**Introduction:** Rapid and reliable neurovascular imaging is critical for time-sensitive diagnosis in acute cerebrovascular disorders, yet conventional magnetic resonance imaging (MRI) workflows remain constrained by acquisition speed, motion sensitivity, and limited integration of physiological context. We introduce the Intelligent Neurovascular Imaging Engine (INIE), a sensor-informed, topology-aware framework that jointly optimizes accelerated data acquisition, physics-grounded reconstruction, and cross-scale physiological consistency. **Methods:** INIE combines adaptive sampling, structured low-rank (Hankel) priors, and topology-preserving objectives with multimodal physiological sensors and scanner telemetry, enabling phase-consistent gating and confidence-weighted reconstruction under realistic operating conditions. The framework was evaluated using synthetic phantoms, a translational porcine stroke recanalization model with repeated measures, and retrospective human datasets. Across $N_{\mathrm{runs}}=120$ acquisition–reconstruction runs derived from $N_{\mathrm{animals}}=18$ pigs with animal-level train/validation/test separation, performance was assessed using image quality, topological fidelity, and cross-modal consistency metrics. Multiple-comparison control was performed using Bonferroni/Holm–Bonferroni procedures. **Results:** INIE achieved acquisition acceleration exceeding 70% while maintaining high reconstruction fidelity (PSNR ≈35–36 dB, SSIM ≈0.90–0.92). Topology-aware analysis showed an approximately twofold reduction in Betti number deviation relative to baseline accelerated methods. Cross-modal validation in a PET subset demonstrated strong agreement between MRI-derived perfusion parameters and metabolic markers (Pearson r≈0.9). INIE improved large-vessel occlusion detection accuracy to approximately 93% and reduced automated time-to-decision to under three minutes. **Conclusions:** These results indicate that sensor-informed, topology-aware, closed-loop imaging improves the reliability and physiological consistency of accelerated neurovascular MRI and supports faster, more robust decision-making in acute cerebrovascular imaging workflows.

## 1. Introduction

Hyperacute ischemic stroke remains a leading cause of death and long-term disability worldwide, where rapid diagnosis and treatment are decisive determinants of outcome. The clinical imperative is well known: delays of only minutes can translate into substantial irreversible neuronal loss, making time-to-decision a practical bottleneck in triage and endovascular intervention. Multimodal imaging—MR angiography (MRA), diffusion/perfusion MRI, and positron emission tomography (PET)—can provide complementary vascular and physiological information (occlusion site, collateral routes, tissue at risk, and metabolic state), yet these modalities remain constrained by acquisition time, motion sensitivity, and reconstruction strategies that typically optimize pixelwise fidelity rather than decision-relevant structure.

A central but under-addressed vulnerability is the preservation of vascular topology. In neurovascular imaging, small reconstruction errors concentrated along thin vessels can disconnect distal branches or fabricate spurious connections, producing fragmented collaterals or hallucinated loops. Such topology distortions are not merely cosmetic: they can bias downstream estimates of perfusion mismatch and collateral status and, consequently, influence the decision to proceed with thrombectomy or adjust recanalization strategy. Therefore, beyond classical fidelity metrics, the reconstruction objective should respect clinically meaningful invariants such as connectivity, bifurcations, and collateral pathways.

Over the past two decades, compressed sensing (CS) [1,2], partial Fourier (PF) reconstruction [3], and variable-density or Poisson-disc sampling strategies [4,5] have progressively accelerated acquisition and improved image quality. However, these advances largely remain modular: CS accelerates acquisition but does not explicitly prioritize topology; deep SR can enhance apparent detail but risks hallucination under distribution shift; topology-aware reconstruction losses constrain structure but typically do not inform what to acquire next; and translational validations are often retrospective and not coupled to acquisition-policy design. Consequently, a methodological gap persists: there is no unified paradigm that couples adaptive acquisition, physics-grounded reconstruction, topology preservation, uncertainty quantification, and translational validation into a single closed-loop imaging system.

To address this gap, we propose the Intelligent Neurovascular Imaging Engine (INIE), a task-driven MR–PET imaging framework that reframes neurovascular imaging as an intelligent sensing problem. INIE integrates (i) accelerated acquisition using Poisson-disc initialization, PF constraints, and uncertainty-aware sampling; (ii) stable reconstruction with structured priors (including Hankel-structured low-rank constraints) and data-consistency enforcement; and (iii) topology-preserving super-resolution to improve reliability of vascular graphs. Crucially, INIE uses an uncertainty signal in topology space (rather than solely pixel space) to guide sampling decisions, prioritizing information that is most relevant to vascular connectivity and clinical interpretability.

INIE is evaluated across synthetic phantoms, heterogeneous retrospective human data, and a clinically relevant porcine stroke model. Importantly, the porcine experiments are organized as repeated acquisition–reconstruction runs per animal (e.g., different acceleration factors and timepoints). To avoid information leakage, all learning-based components are trained and tuned with animal-level splits (no animal contributes to more than one split), and inferential statistics are reported with hierarchical/mixed-effects modeling that accounts for within-animal correlation. Moreover, we explicitly separate scanner acquisition time from computation time and report time-to-decision in a workflow-defined manner, reflecting the practical conditions under which the system could support acute decision-making.

### Contributions

The main contributions of this work are (i) a unified closed-loop framework coupling accelerated acquisition, physics-grounded reconstruction, topology-preserving SR, and uncertainty-driven sampling; (ii) a topology-centric uncertainty signal that links acquisition choices to clinically meaningful vascular graph stability; (iii) a reproducible evaluation design with leakage-proof animal-level splits, comprehensive baselines, and mixed-effects statistics; and (iv) multi-scale validation on phantoms, retrospective human scans, and a translational porcine stroke model with cross-modal physiological benchmarking.

This paper is organized as follows. Section 2 reviews related work and motivates the research gap addressed by INIE. Section 3 details the INIE methodology, including acquisition policy, reconstruction, topology components, and uncertainty modeling. Section 4 describes evaluation metrics, baselines, and fairness controls. Section 5 presents ablations and key results, and Section 6 reports comprehensive validation across fidelity, topology, perfusion/PET concordance, and decision-oriented endpoints. Section 7 discusses implications, translational grounding, limitations, and future work. Finally, Section 8 concludes the paper.

In sum, INIE aims to move neurovascular imaging from a descriptive modality toward an adaptive, topology-aware, decision-oriented system. By embedding uncertainty-aware acquisition and translational validation into the system design, it establishes a principled foundation for next-generation stroke imaging pipelines.

## 2. Related Work and Research Gap

Hyperacute neurovascular imaging requires a rare combination of properties: (i) aggressive acceleration, (ii) high-fidelity depiction of distal and collateral vessels, and (iii) reliability guarantees that are meaningful for clinical decisions (e.g., the connectivity of the vascular tree rather than pixelwise similarity). Existing advances address parts of this problem, but typically in isolation. This section summarizes four converging research directions and motivates the integrated design of INIE.

### 2.1. Accelerated Sampling and Reconstruction

Compressed sensing (CS) and partial Fourier methods enable reduced acquisition by exploiting sparsity and Fourier-domain symmetries. In MRI, undersampled measurements can be expressed as $y=F_{M}x+\eta$, where $F_{M}$ is the Fourier encoding operator restricted to a sampling mask M and η denotes noise [6]. Classical CS reconstructions solve

(1) $$\hat{x}=\arg\min_{x}\frac{1}{2}∥F_{M}{x-y∥}_{2}^{2}+\lambda {∥\Psi x∥}_{1},$$

with Ψ a sparsifying transform [7]. In neurovascular settings, the practical limitation is that pixelwise recovery does not necessarily imply preservation of vascular connectivity: thin vessels and distal branches occupy a small fraction of voxels, and small structured errors can break continuity or distort bifurcations while leaving global metrics seemingly adequate.

### 2.2. Super-Resolution and Structured Priors

Super-resolution (SR) seeks to recover high-resolution structure from degraded or intentionally reduced data [8,9]. Deep SR models (CNN/GAN families) can improve perceived sharpness but may introduce clinically unsafe hallucinations when trained with generic perceptual or adversarial losses, particularly under distribution shift [10,11]. Physics-grounded constraints (data consistency) reduce instability, and structured priors further improve robustness under extreme undersampling. Hankel-structured low-rank models provide stability by converting local redundancy into a matrix-completion view of the inverse problem [12,13]. However, most SR pipelines still prioritize pixelwise losses and do not explicitly optimize topology or downstream decision stability. In the MRI context, super-resolution and extreme undersampling have been addressed using combinations of compressed sensing and reconstruction priors, including highly sparse input regimes and hybrid SR–CS formulations [14,15,16].

### 2.3. Topology-Aware Objectives

For stroke triage, the decision-relevant invariant is often the vascular graph: continuity, bifurcations, and collateral pathways. Topology-aware losses, therefore, complement pixelwise losses by penalizing changes in connectivity and vessel graph structure, including approaches based on persistent homology and differentiable topology surrogates [17,18,19,20,21,22]. This direction is promising, but in most existing work, topology appears only as an auxiliary reconstruction regularizer, while sampling remains fixed. Moreover, topology metrics can be sensitive to segmentation/thresholding choices; therefore, reproducible evaluation requires explicit reporting of filtration choices, graph extraction procedures, and sensitivity analyses, which we provide in later sections.

### 2.4. Cross-Modal and Translational Validation

Multimodal validation using perfusion imaging, PET metabolic measures, micro-CT/casting, and histology can strengthen claims of physiological fidelity beyond pixelwise similarity. Large-animal models provide controlled conditions and more human-like neurovascular anatomy than rodent models, enabling meaningful evaluation of vascular topology and collateral behavior. However, validation is commonly performed after acquisition with fixed protocols, such that reconstruction and sampling are not jointly optimized under translational constraints. In addition, cross-modal comparisons (e.g., MRI–PET correlations) require careful reporting of tracer protocols, reconstruction settings, registration QC, and sensitivity to misalignment, because modest misregistration can inflate or deflate correlation-based metrics.

### 2.5. Research Gap

The unresolved gap is the absence of a closed-loop framework that simultaneously (i) adapts sampling to reduce *topology-relevant* uncertainty, (ii) enforces physics-grounded reconstruction with stable priors and controlled hallucination risk, and (iii) is evaluated with translational protocols where topology errors can be connected to perfusion and physiological proxies. INIE is designed to address this gap by coupling accelerated acquisition, topology-preserving reconstruction, uncertainty-aware sampling, and cross-scale validation within a single task-driven pipeline.

## 3. The Intelligent Neurovascular Imaging Engine (INIE)

The Intelligent Neurovascular Imaging Engine (INIE) reformulates neurovascular imaging as a closed-loop, topology-aware inference system in which acquisition, reconstruction, uncertainty estimation, and downstream physiological interpretation are explicitly coupled. In contrast to conventional pipelines that rely on static sampling patterns and optimize primarily pixelwise fidelity, INIE adapts its sensing strategy to minimize uncertainty in clinically critical vascular connectivity and collateral structure [23,24].

At the system level, INIE treats the imaging chain as an intelligent sensor–inference loop whose goal is not merely to reconstruct an image but to acquire the *right measurements*—those that stabilize decision-relevant vascular topology and reduce ambiguity in biomarkers (e.g., LVO, collateral grade, mismatch surrogates). Importantly, the term “porcine experiments” used throughout the paper refers to *acquisition–reconstruction runs*. Multiple runs are typically obtained per animal (e.g., timepoints, acceleration factors, or perturbation conditions). To avoid information leakage, all learning-based components are trained and tuned using *animal-level* splits, and statistical inference is performed with hierarchical models that account for within-animal correlation (Section 5.1).

### 3.1. Closed-Loop Formulation and System Architecture

INIE formulates neurovascular imaging as a closed-loop sensing and inference problem, in which data acquisition, image reconstruction, topology extraction, and uncertainty estimation are iteratively coupled. Unlike conventional pipelines based on a fixed sampling mask and a single reconstruction step, INIE continuously adapts its acquisition strategy based on the evolving state of the reconstruction and the estimated reliability of *clinically relevant* structures.

Let $x\in C^{N}$ denote the unknown neurovascular image (or multi-channel image) to be reconstructed, where *N* is the number of voxels and complex values arise naturally from Fourier-encoded modalities such as MRI. The measurement process is described by a modality-specific forward operator $F_{M}$ parameterized by a sampling mask or acquisition policy M. In MRI, $F_{M}$ corresponds to masked Fourier encoding; in PET, it corresponds to projection/sinogram formation and reconstruction operators; and in hybrid MR–PET settings, $F_{M}$ can denote a block-structured combination with modality-specific masks.

At iteration *t*, the system has acquired a sequence of measurements governed by sampling actions ${\left\{M_{\tau }\right\}}_{\tau =1}^{t}$:

(2) $$Y_{t}={\left\{F_{M_{\tau }}\left(x\right)\right\}}_{\tau =1}^{t}.$$

In a closed-loop setting, $M_{\tau }$ is not fixed a priori but selected adaptively from feasible hardware-constrained candidates based on feedback from previous reconstructions.

Given the measurement history $Y_{t}$, the current estimate ${\hat{x}}_{t}$ is obtained via a reconstruction operator

(3) $${\hat{x}}_{t}=R_{\phi }\left(Y_{t}\right),$$

where $R_{\phi }$ denotes a parameterized reconstruction engine and ϕ collects learnable weights (for unrolled/deep modules), regularization strengths, and algorithmic hyperparameters. The operator $R_{\phi }$ enforces complementary constraints: (i) *data consistency* with all acquired measurements under $F_{M_{\tau }}$; (ii) *structured Hankel low-rank priors* stabilizing recovery under severe undersampling; (iii) *partial Fourier constraints* exploiting conjugate symmetry and phase consistency; and (iv) *topology-aware regularization* penalizing disruptions of vascular connectivity (broken branches, spurious loops).

The reconstruction ${\hat{x}}_{t}$ is then analyzed to extract higher-order representations (vascular graphs and topological descriptors) and to estimate uncertainty *at the structure level*. This uncertainty drives the selection of the next acquisition action $M_{t+1}$, closing the loop between reconstruction and sensing.

#### Implementation Scope: Scanner-Synchronized Workflow

INIE is implemented as a scanner-synchronized pipeline: acquisition proceeds on the scanner with feasible, pre-validated candidate masks/trajectories, while iterative reconstructions, topology analysis, and decision outputs are produced on a dedicated workstation in near-real time. Unless explicitly stated otherwise, “time-to-decision” refers to *acquisition time plus automated computation* (Section 6) and does not include human interpretation time.

**Figure 1:**
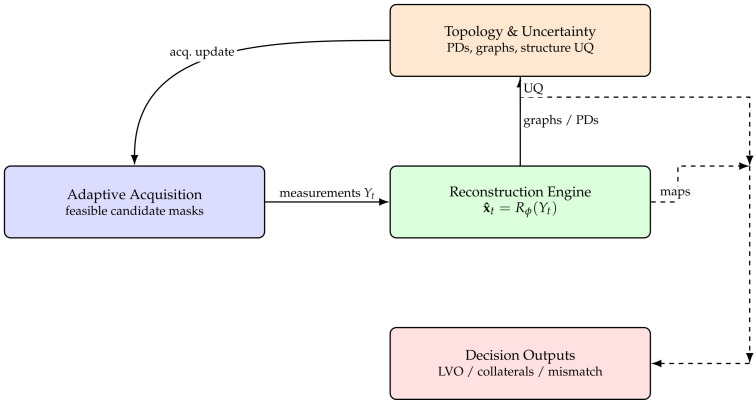
Closed-loop architecture of the Intelligent Neurovascular Imaging Engine (INIE). Acquisition, reconstruction, and topology-aware uncertainty analysis are coupled through explicit feedback, transforming imaging into an adaptive, decision-driven sensing process. Solid arrows denote the forward dataflow across the pipeline, whereas dashed arrows denote auxiliary outputs to the decision module (maps and uncertainty summaries). The curved solid arrow indicates feedback from topology and uncertainty analysis to the acquisition module, updating the sampling policy. Color coding groups functional blocks: blue—acquisition, green—reconstruction, orange—topology and uncertainty analysis, and red—decision outputs. Implementation is scanner-synchronized: acquisition occurs on-scanner, while reconstruction, analysis, and outputs are generated on a dedicated workstation.

From a control perspective, $F_{M}$ acts as the plant, $R_{\phi }$ as the state estimator, and topology-aware uncertainty as a feedback signal. This transforms imaging from a static feed-forward procedure into an active, information-driven process in which each new measurement is chosen to maximize impact on clinically critical structures rather than global pixelwise error alone. The architecture is illustrated in Figure 1.

This architecture distinguishes INIE from existing accelerated imaging approaches by making *structure-level uncertainty*—rather than sampling density or pixel error alone—the primary driver of measurement selection.

### 3.2. Topology-Aware Uncertainty Estimation

A defining component of INIE is the explicit estimation of uncertainty at the level of vascular topology, rather than solely at the level of individual pixels or voxels. This design reflects the clinical reality that decision-making in neurovascular disease depends on the integrity of vessel connectivity, collateral pathways, and branching patterns, which are not adequately captured by conventional pixelwise uncertainty maps.

Starting from the reconstructed image ${\hat{x}}_{t}$, a vascular representation $T({\hat{x}}_{t})$ is extracted using a reproducible processing pipeline comprising vesselness filtering, segmentation, and skeletonization. The resulting abstraction can be interpreted as a graph of the vascular tree (nodes: bifurcations/terminations; edges: vessel segments). Because topology computations can depend on segmentation thresholds, INIE evaluates topology stability across a narrow threshold band and reports sensitivity analyses (Section 3.3).

To quantify the reliability of vascular connectivity in a scale-aware and noise-robust manner, INIE employs persistent homology. Persistent homology characterizes the topological structure of $T({\hat{x}}_{t})$ across scales, capturing features such as connected components (Betti number $\beta_{0}$) and loops/collateral circuits (Betti number $\beta_{1}$) [17]. These descriptors are insensitive to small geometric perturbations while remaining sensitive to genuine connectivity changes.

Topological uncertainty is defined as the instability of persistent features under stochastic perturbations of the reconstruction process. Specifically, multiple stochastic realizations ${\hat{x}}_{t}^{\left(k\right)}$ are generated (Monte Carlo dropout, randomized regularization, or controlled noise injection within $R_{\phi }$). For each realization, a persistence diagram $\mathrm{PD}({\hat{x}}_{t}^{\left(k\right)})$ is computed and compared with the reference diagram $\mathrm{PD}({\hat{x}}_{t})$ obtained from the mean/deterministic reconstruction. The topology-aware uncertainty is

(4) $$U\left({\hat{x}}_{t}\right)=E\;\left[d_{\mathrm{Wass}}\;\left(\mathrm{PD}\left({\hat{x}}_{t}^{\left(k\right)}\right),\mathrm{PD}\left({\hat{x}}_{t}\right)\right)\right],$$

where $d_{\mathrm{Wass}}\left(\cdot ,\cdot \right)$ is the Wasserstein distance between persistence diagrams and the expectation is taken over stochastic realizations indexed by *k*. This measure directly captures clinically meaningful failure modes that can be invisible to pixelwise variance, e.g., intermittent disappearance of a collateral branch or stochastic emergence of a spurious loop [18].

**Figure 2:**
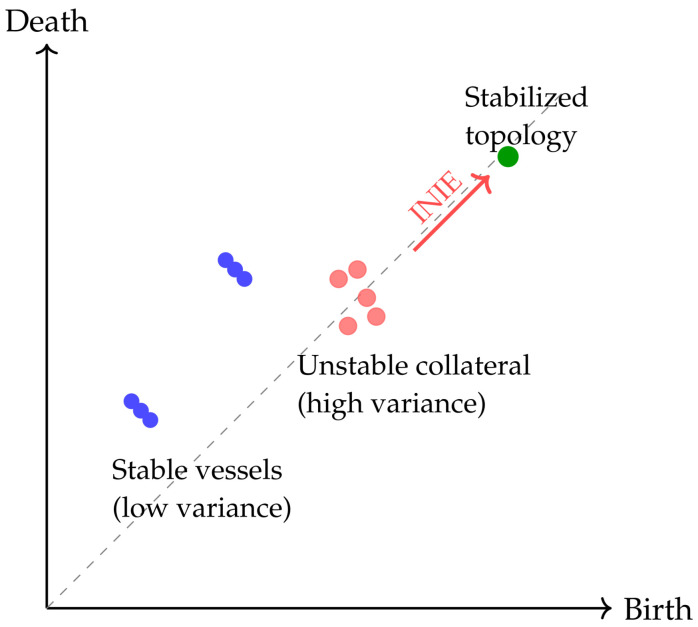
Topology-aware uncertainty in persistence space. Stable vascular features form compact clusters far from the diagonal. Unstable collateral loops manifest as dispersed persistence points across stochastic reconstructions. INIE targets these regions, reducing topological variance and stabilizing clinically relevant connectivity.

Figure 2 illustrates the intuition in persistence space: stable vascular features cluster far from the diagonal, whereas unstable collateral pathways generate dispersed point clouds. Within INIE, regions associated with high topological uncertainty are targeted by adaptive acquisition, reducing variance and converting unstable features into consistent, long-lived structures that are more reliable for downstream physiological interpretation.

### 3.3. Topology Metrics and Implementation Details

This subsection details the practical computation of vascular topology descriptors used throughout the INIE framework, including graph extraction, filtration construction, persistent homology computation, and derived stability metrics.

#### 3.3.1. Vascular Graph Extraction

Starting from the reconstructed angiographic volume ${\hat{x}}_{t}$, vessels are enhanced using a multi-scale vesselness filter, followed by threshold-based segmentation and 3D skeletonization. The resulting centerline representation is converted into a graph G=(V,E), where vertices correspond to bifurcations or terminal points and edges correspond to vessel segments. Each edge is annotated with geometric attributes (length, mean radius) and intensity-derived confidence scores. To reduce sensitivity to spurious small branches, components shorter than a fixed physical length (2–3 mm in the current implementation) are pruned prior to topological analysis.

#### 3.3.2. Filtration and Persistence Computation

Persistent homology is computed on a superlevel-set filtration of the vesselness response mapped onto the skeleton graph. Specifically, edge weights are defined from the vesselness or intensity confidence, and a decreasing-threshold filtration is constructed, yielding a nested sequence of graphs. From this filtration, persistence diagrams are computed for $\beta_{0}$ (connected components) and $\beta_{1}$ (loops/collateral circuits) using a standard cubical/graph-based persistence backend. Birth and death times, therefore, correspond to the appearance and disappearance of connected components and cycles as the confidence threshold is varied.

#### 3.3.3. Topology Deviation and Stability Metrics

Topology deviation is quantified as the Wasserstein distance between the persistence diagram of a candidate reconstruction and that of a high-quality reference reconstruction obtained from densely sampled data. Persistence stability is defined as one minus the normalized average Wasserstein distance across stochastic reconstructions, such that higher values indicate more stable and reproducible topology. Branch completeness is computed as the fraction of reference graph edges that can be matched (within a spatial tolerance) to edges in the reconstructed graph.

#### 3.3.4. Sensitivity to Segmentation and Thresholds

Because topological descriptors depend on the upstream segmentation and skeletonization pipeline, we evaluate stability across a range of reasonable vesselness thresholds and report persistence-based distances, which are less sensitive to single-threshold failures than hard graph comparisons. In practice, we observe that topology-aware methods can still fail in edge cases (e.g., extremely low SNR or severe motion), motivating the combined use of persistence stability, branch completeness, and visual inspection in the evaluation.

#### 3.3.5. Role Within INIE

These topology metrics serve two roles in the framework: (i) they define the topology-aware uncertainty measure used to guide adaptive acquisition, and (ii) they provide structure-level evaluation criteria that complement pixelwise image-quality metrics in the Results section.

### 3.4. Adaptive Acquisition Driven by Uncertainty Reduction

INIE formulates acquisition as an adaptive decision process whose objective is not uniform coverage of measurement space but targeted reduction of topology-aware uncertainty. At each iteration *t*, the acquisition policy evaluates candidate sampling actions and selects the one expected to maximally stabilize vascular connectivity and collateral structure.

Let M denote a feasible sampling mask or trajectory (e.g., a set of *k*-space lines in MRI or a constrained subset of projections in PET). The next acquisition action is chosen as

(5) $$M_{t+1}=\arg\max_{M}E\;\left[U\left({\hat{x}}_{t}\right)-U\;\left(R_{\phi }\;\left(Y_{t}\cup F_{M}x\right)\right)\right],$$

where U(·) is the topology-aware uncertainty measure, $F_{M}$ is the forward operator for the candidate action, and the expectation captures stochasticity in reconstruction and uncertainty estimation. This explicitly prioritizes measurements predicted to resolve ambiguous topological features such as poorly supported distal branches or fragile collateral loops.

Direct evaluation of all possible acquisition actions is computationally intractable. INIE therefore uses a constrained candidate set of trajectories generated under scanner hardware and safety constraints, evaluated using fast surrogate reconstructions that approximate uncertainty reduction without full iterative convergence. This enables near-real-time adaptation within a scanner-synchronized workflow. Poisson-disc initialization enforces incoherent sampling patterns with blue-noise characteristics [4,5], while partial Fourier constraints exploit conjugate symmetry in k-space [3].

To ensure physical realizability and robustness, the policy is constrained by established acquisition principles. Poisson-disc initialization enforces incoherence and prevents large unsampled gaps (supporting stable CS-type recovery) [23]. Partial Fourier constraints exploit conjugate symmetry and promote phase consistency, improving robustness to incomplete sampling and motion [25]. The net effect is a reallocation of measurement density toward regions expected to yield the highest information gain for vascular topology rather than generic image-quality criteria.

**Figure 3:**
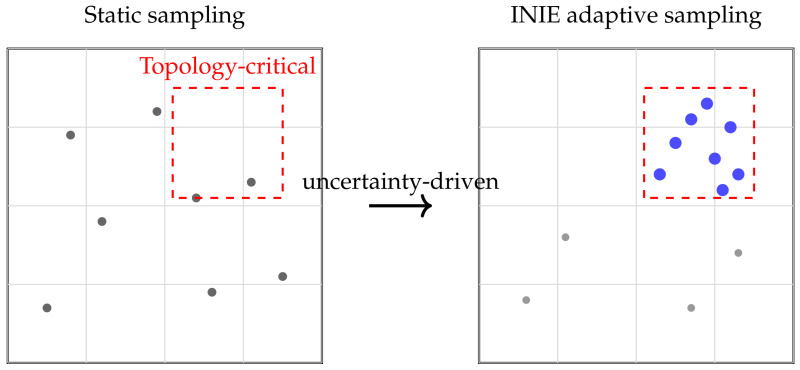
Schematic effect of uncertainty-driven adaptive reallocation of measurements in INIE. (**Left**): static sampling distributes measurements more uniformly, potentially undersampling topology-critical regions. (**Right**): INIE reallocates measurements toward regions associated with high topology uncertainty, increasing sampling density where connectivity is most informative. Black points denote baseline (uniform) sampling locations, blue points denote adaptively added measurements in high-uncertainty regions, and the red dashed box indicates a topology-critical region of interest.

Figure 3 schematically contrasts static sampling with uncertainty-driven adaptive reallocation. In practice, “topology-critical” regions correspond to spatial neighborhoods in image space whose extracted graphs/persistence signatures exhibit high instability; these are mapped to candidate measurement updates via the forward model and a feasibility-constrained action set.

### 3.5. Translational Implementation and Validation

INIE is implemented and validated in a clinically relevant porcine stroke model using multimodal MRI, PET, and angiography, enabling simultaneous assessment of macro-scale vascular topology, meso-scale perfusion dynamics, and micro-scale metabolic viability [16]. In the experimental protocol, each animal can contribute multiple acquisition–reconstruction runs (e.g., across timepoints and acceleration factors), which are treated as repeated measures. Cross-modal analyses use PET primarily as an independent physiological reference, with explicit MR–PET registration QC and sensitivity analyses reported later (Section 5.3). By grounding mathematical objectives in measurable physiological outcomes and enforcing leakage-proof animal-level evaluation, INIE establishes a direct link between sensing strategy and clinically meaningful decision stability.

## 4. Cross-Scale Simulator: Macro–Meso–Micro

Beyond image reconstruction, the Intelligent Neurovascular Imaging Engine (INIE) incorporates a cross-scale simulator that propagates uncertainty from vascular topology to physiological function and tissue outcome proxies. The purpose of this module is twofold: (i) to quantify how reconstruction-induced errors—especially topological inconsistencies in vascular connectivity—affect downstream perfusion and viability estimates; and (ii) to provide a mechanistic, physiology-grounded validation axis that complements pixelwise and topology metrics. This addresses a key translational concern: even small topology errors in thin vessels can produce clinically meaningful distortions in perfusion territories and mismatch surrogates.

To keep the simulator tractable for repeated uncertainty propagation, and ablations, we use a lightweight, hybrid formulation that combines graph-based hemodynamic surrogates (macro), tissue-level transport dynamics (meso), and a compact energy-based viability proxy (micro). Unless otherwise stated, all simulator parameters are set *a priori* and are not tuned on test data.

### 4.1. Macro Scale: Vascular Graph Representation and Topology Descriptors

At the macro scale, the reconstructed angiographic volume ${\hat{x}}_{t}$ is transformed into a vascular graph G=(V,E) via vesselness filtering, segmentation, skeletonization, and graph extraction. Each vertex v∈V corresponds to a bifurcation, junction, or termination, and each edge e∈E represents a vessel segment. Segment attributes include diameter $d_{e}$, length $\ell_{e}$, and an effective conductance term $g_{e}$ estimated from geometry:

(6) $$g_{e}\propto \frac{d_{e}^{\;4}}{\ell_{e}+\epsilon },$$

motivated by Poiseuille-type scaling. This parameterization enables a robust mapping from topology to flow capacity without requiring full computational fluid dynamics.

Topological fidelity at the macro scale is assessed using (i) graph metrics (connectivity, degree distribution, shortest-path redundancy, branch completeness) and (ii) persistent homology descriptors. In particular, Betti numbers $\beta_{0}$ and $\beta_{1}$ quantify connected components and loops/collateral circuits, providing a scale-aware characterization of vascular connectivity [17]. Deviations in these descriptors across stochastic reconstructions reflect topological uncertainty introduced by acquisition/reconstruction.

#### Topology Uncertainty Summary Used by the Simulator

For uncertainty propagation we summarize topology instability by a scalar $U_{\mathrm{macro}}$ that combines diagram-level variation and graph-level fragmentation:

(7) $$U_{\mathrm{macro}}\;=\;w_{1}\;E_{k}\;\left[d_{\mathrm{Wass}}\;\left(\mathrm{PD}\left({\hat{x}}^{\left(k\right)}\right),\mathrm{PD}\left(\hat{x}\right)\right)\right]\;+\;w_{2}\;\left(1-\mathrm{BC}\right)\;+\;w_{3}\;\Delta \beta ,$$

where BC is branch completeness, Δβ is Betti deviation (Section 3.3), and $w_{1},w_{2},w_{3}$ are fixed weights. This composite signal makes uncertainty propagation sensitive to clinically relevant failure modes (e.g., missing distal branches or spurious loops) rather than being dominated by global pixel noise.

### 4.2. Meso Scale: Perfusion and Transport Modeling

The meso-scale module maps macro-scale vascular structure to tissue-level perfusion and tracer transport. We use an advection–diffusion–reaction formulation for a concentration field c(v,t) defined over tissue locations *v*:

(8) $$\frac{\partial c}{\partial t}\left(v,t\right)+\nabla \;\cdot \;\left(u(v)\;c(v,t)\right)=D\nabla^{2}c\left(v,t\right)-\lambda c\left(v,t\right)+S\left(v,t\right).$$

Here, u(v) is an effective velocity field derived from the vascular graph and its conductances, *D* captures microvascular dispersion, λ models clearance/decay, and S(v,t) represents injection or tracer input. In practice, u(v) is computed using a graph-to-field mapping: flow along each graph edge is estimated from conductance-weighted pressures, and tissue velocities are obtained by spatially smoothing edge-wise flow contributions into a voxel grid. This yields a stable surrogate that is sensitive to topology but computationally inexpensive.

#### 4.2.1. Topology-to-Perfusion Sensitivity

Errors at the macro scale perturb the velocity field u(v) and thus propagate to meso-scale perfusion estimates. Missing collateral connections tend to reduce flow redundancy and increase transit delays, whereas spurious loops can introduce unphysical shortcuts. These effects manifest as biased perfusion territories and distortions in perfusion-derived parameters (e.g., delayed arrival time, elevated Tmax, and altered CBF/CBV proxies). This sensitivity makes the meso module a critical bridge between topology fidelity and physiological validity.

#### 4.2.2. Perfusion Endpoints

From c(v,t), we compute compact perfusion descriptors used for validation and decision tasks, including (i) peak concentration (CBV proxy), (ii) time-to-peak/Tmax proxies, and (iii) area-under-curve (CBF proxy). These are compared against reference perfusion maps and cross-modal physiological measures in Section 6.5.

### 4.3. Micro Scale: Neuronal Viability and Tissue Outcome Proxy

At the micro scale, meso-scale perfusion is linked to tissue viability through an energy-based proxy model. Each voxel (tissue location) *v* is assigned an energy-like state E(v,t):

(9) $$\begin{array}{cc} \frac{dE}{dt}\left(v,t\right) & =\alpha \;\Phi (v)-\beta \;\Gamma (v,t)-\gamma E(v,t), \end{array}$$

(10) $$\begin{array}{cc} P_{\mathrm{survival}}\left(v,t\right) & =\sigma \;\left(E(v,t)-\theta \right), \end{array}$$

where Φ(v) denotes perfusion-derived supply extracted from the meso module, Γ(v,t) summarizes demand/stress (e.g., metabolic load or excitotoxicity proxy), α,β,γ are fixed scaling parameters, and σ(·) is the logistic function. In practice, Γ(v,t) is modeled as a bounded function of time and local perfusion deficit to avoid unrealistic divergence and to preserve interpretability for ablations.

The survival probability $P_{\mathrm{survival}}\left(v,t\right)$ serves as a voxel-wise outcome proxy that can be compared against independent references in experimental data, including PET-derived metabolic markers (e.g., FDG uptake and, where available, oxygen extraction/metabolic surrogates) and histological outcome measures. Although simplified, this model captures the essential monotonic coupling between perfusion adequacy and tissue fate, making it suitable for large-scale uncertainty propagation.

### 4.4. Uncertainty Propagation Across Scales

A central function of the simulator is to propagate macro-level topology uncertainty to downstream physiological predictions. Let $U_{\mathrm{macro}}$ denote topology uncertainty (Equation (7)). This induces variability in meso-scale perfusion endpoints and, consequently, in micro-scale survival estimates. We quantify uncertainty at each scale as the empirical variance across repeated stochastic reconstruction–simulation cycles:

(11) $$\begin{array}{cc} U_{\mathrm{meso}} & ={\mathrm{Var}}_{k}\;\left(P\left({\hat{x}}^{\left(k\right)}\right)\right), \end{array}$$

(12) $$\begin{array}{cc} U_{\mathrm{micro}} & ={\mathrm{Var}}_{k}\;\left(P_{\mathrm{survival}}^{\left(k\right)}\right), \end{array}$$

where P(·) denotes the mapping from reconstruction to perfusion endpoints via Equation (8). For compact reporting, we summarize micro uncertainty by the mean voxel-wise variance in the at-risk territory:

(13) $${\bar{U}}_{\mathrm{micro}}\;=\;\frac{1}{|\Omega_{\mathrm{risk}}|}\sum_{v\in \Omega_{\mathrm{risk}}}{\mathrm{Var}}_{k}\;\left(P_{\mathrm{survival}}^{\left(k\right)}\left(v\right)\right).$$

#### Physiology-Aware Ablations and Validation

This framework enables systematic ablations: acquisition or reconstruction strategies that reduce $U_{\mathrm{macro}}$ should yield measurable reductions in $U_{\mathrm{meso}}$ and ${\bar{U}}_{\mathrm{micro}}$, and improved agreement with independent perfusion and metabolic references. In Section 6.5, we report (i) correlations between predicted and reference perfusion endpoints, (ii) cross-modal agreement with PET metrics, and (iii) infarct/mismatch proxy errors. Importantly, because repeated runs are nested within animals, inferential statistics for these endpoints are performed with mixed-effects models (Section 5.1) to avoid inflated evidence due to within-animal correlation.

By explicitly linking reconstruction uncertainty to physiological and biological outcome proxies, the cross-scale simulator elevates INIE from an image-centric framework to a decision-aware system: it evaluates not only how images look or whether topology is preserved, but also how imaging-level errors propagate into clinically meaningful predictions.

### 4.5. Simulator Parameterization and Numerical Settings (Reproducibility)

To support reproducibility, Table 1 reports the fixed numerical parameters used across all experiments. These values are chosen to produce physiologically plausible dynamics and stable numerical integration rather than to match any single dataset. All parameters were fixed using training/validation animals only, and the same settings were used for the independent test animals.

## 5. Evaluation Protocol

The proposed Intelligent Neurovascular Imaging Engine (INIE) is evaluated using a multi-level protocol aligned with the closed-loop and cross-scale design of the framework. Validation is not limited to conventional image-fidelity measures; instead, it spans (i) macro-scale vascular topology and decision endpoints, (ii) meso-scale perfusion/transport consistency, and (iii) micro-scale metabolic/tissue outcome proxies. The protocol is designed to be (a) leakage-proof at the animal level, (b) statistically valid under repeated measures (multiple runs per animal), and (c) reproducible via explicit preprocessing, baseline, and QC specifications.

### 5.1. Study Design, Sample Size, Data Splits, and Statistical Analysis

#### 5.1.1. Unit of Evidence (Animals vs. Runs)

The dataset contains $N_{\mathrm{runs}}=120$ **porcine acquisition–reconstruction runs**. These runs originate from $N_{\mathrm{animals}}=18$ pigs (mean ± SD: 6.7±1.4 runs/animal). Runs from the same animal are correlated (shared anatomy, protocol, and timepoints) and therefore are **not treated as independent** for inferential statistics.

#### 5.1.2. Leakage-Proof Splits (Animal-Level)

All learning-based components were trained and tuned using **animal-level splits** (no animal contributes to more than one split). The cohort was divided into (i) training/validation: twelve animals (80 runs) and (ii) independent test: six animals (40 runs), held out until final evaluation. Within the 12 development animals, 5-fold cross-validation at the animal level was used for hyperparameter selection and ablation studies.

#### 5.1.3. Primary and Secondary Endpoints

Primary endpoints were (i) topology preservation (Betti deviation, persistence stability, and branch completeness) and (ii) decision-oriented performance (LVO detection, collateral scoring, and mismatch AUC). Secondary endpoints included pixelwise fidelity (PSNR, SSIM, NMSE, LPIPS), cross-modal physiological consistency (MRI–PET correlation, SUV recovery), runtime/time-to-decision decomposition, and robustness under perturbations (motion, SNR reduction, and MR–PET misregistration).

#### 5.1.4. Statistical Inference with Repeated Measures

To avoid inflated evidence due to repeated runs per animal, inferential comparisons are performed using **mixed-effects models** with an animal-specific random intercept: (i) linear mixed models for continuous outcomes (SSIM, Betti deviation, correlations, perfusion errors, runtime); (ii) generalized mixed models (logit link) for binary endpoints (LVO); and (iii) ordinal/multinomial mixed models for collateral grade where applicable. We report effect sizes (INIE minus best baseline), 95% confidence intervals (CI), and multiple-comparison adjusted *p*-values (Holm–Bonferroni [26], α=0.05). Run-level mean ± SD is reported descriptively for completeness, but **statistical significance is at the animal-level**.

### 5.2. Datasets, Inclusion/Exclusion, and Experimental Model

Experiments are conducted using a clinically relevant porcine middle cerebral artery occlusion (MCAO) model with controlled recanalization. The porcine cerebrovascular system resembles human cerebral anatomy in vessel caliber, branching patterns, and collateral organization, supporting translational evaluation of topology and downstream physiology.

Imaging protocols include time-of-flight MR angiography (TOF-MRA) for macro-scale vascular structure, diffusion-weighted imaging (DWI) and ADC maps for tissue injury, dynamic perfusion MRI for meso-scale hemodynamics, and PET metabolic imaging in a subset. Ground-truth references include ex vivo vascular casting, micro-CT (subset), and histology (subset), enabling independent validation of topology and tissue fate without relying on a single modality as truth.

#### 5.2.1. Inclusion/Exclusion and QC

Animals/runs were included if (i) MCAO was successfully induced and verified by angiography/MRA; (ii) core MR sequences were acquired with sufficient quality for registration; (iii) recanalization status was documented; and (iv) PET (when present) passed QC. Exclusion criteria included off-target occlusion, major acquisition failure, non-correctable motion, or registration QC failure. The summary is reported in Table 2.

#### 5.2.2. Cross-Modal PET Subset (Reference-Only Unless Stated)

PET was available in 10/18 animals (52/120 runs). Unless explicitly stated, PET was used as an **independent reference** for validation (SUV recovery, correlation with perfusion/metabolic proxies) and was not provided as an input to single-modality baselines. MR–PET registration used rigid+affine alignment with mutual-information optimization and visual QC; sensitivity to misregistration is evaluated in the robustness analysis.

### 5.3. PET Acquisition, Reconstruction, and Registration

Positron emission tomography (PET) data were acquired in a subset of animals to provide independent metabolic reference measurements for cross-modal validation. Two tracers were used in separate sessions: an oxygen extraction fraction (OEF) tracer and a cerebral metabolic rate of glucose (CMRglc) tracer, following standard experimental neuroimaging protocols. PET acquisition was performed under stable physiological conditions, with tracer injection and uptake times matched across animals as closely as possible.

PET images were reconstructed using a standard iterative ordered-subsets expectation maximization (OSEM) algorithm with corrections for attenuation, scatter, and randoms, as provided by the vendor reconstruction software. The resulting PET volumes were resampled to the MRI spatial grid using rigid registration based on mutual information. Registration quality was assessed visually and by inspecting landmark alignment in vascular and cortical structures; datasets with gross misregistration were excluded from cross-modal analysis.

Importantly, PET data were used *only* for evaluation and validation of physiological consistency and tissue outcome proxies and were never provided as input to the INIE reconstruction or acquisition policy. Cross-modal metrics such as perfusion–metabolism correlation and SUV recovery, therefore, reflect agreement between independently reconstructed modalities rather than information leakage between inputs.

To assess sensitivity to residual misregistration, additional experiments were performed with synthetic rigid perturbations (0–3 mm translations) applied to the PET volumes prior to metric computation. The resulting variation in correlation and SUV recovery is reported in the robustness analysis, providing context for the stability of cross-modal validation results.

### 5.4. Evaluation Tasks Across Scales

Tasks correspond to the macro/meso/micro components of the INIE cross-scale module:

#### 5.4.1. Macro Scale

(i) Large-vessel occlusion (LVO) detection (binary; side when applicable); (ii) collateral circulation assessment (ordinal bins; evaluated via F1 and AUC where applicable); (iii) topology preservation via persistent homology and graph metrics (Betti deviation, persistence stability, branch completeness).

#### 5.4.2. Meso Scale

Perfusion mismatch estimation and transport-related quantities derived from dynamic imaging, including Tmax delay error and correlation of CBF/CBV proxies with reference perfusion.

#### 5.4.3. Micro Scale

PET SUV recovery (subset) and neuronal survival/tissue outcome proxy agreement (subset), evaluated against metabolic imaging and histology where available.

### 5.5. Quantitative Metrics and Reporting

Image fidelity is assessed using PSNR, SSIM, NMSE, and LPIPS. Topology is evaluated using Betti deviation, branch completeness and persistence-diagram stability. Cross-modal physiological plausibility uses MRI–PET correlation and SUV recovery (subset). Decision endpoints include LVO accuracy, collateral F1, mismatch AUC, and time-to-decision.

All metrics are reported as run-level mean ± SD for descriptive completeness, and as **animal-level mixed-effects estimates with 95% CI** for inferential conclusions (Table 3).

## 6. Results

The Intelligent Neurovascular Imaging Engine (INIE) was evaluated using synthetic phantoms, a translational porcine MCAO model, and a heterogeneous retrospective human cohort. Unless otherwise specified, descriptive values are reported over $N_{\mathrm{runs}}=120$ porcine runs, with inferential statistics computed at the animal level ($N_{\mathrm{animals}}=18$) using mixed-effects models (Section 5.1). Where cross-modal PET endpoints are reported, they use the PET subset (10 animals; 52 runs). All multiple comparisons use Holm–Bonferroni correction (α=0.05).

### 6.1. Runtime and Computational Performance

Runtime was evaluated to assess the feasibility of scanner-synchronized, near-real-time decision support. All experiments were performed on a workstation equipped with an NVIDIA RTX A6000 GPU (24 GB VRAM; NVIDIA Corporation, Santa Clara, CA, USA) and a 16-core CPU.

Reconstruction used a hybrid GPU–CPU pipeline: data consistency and Hankel operations were executed on the GPU, while topology extraction and graph operations were executed asynchronously on the CPU.

Across 120 runs, the average runtime per adaptive reconstruction iteration was 0.42±0.08 s. Each scan required 6–8 adaptive iterations, yielding an average end-to-end reconstruction time of 2.6±0.4 s per volume (computation only). To avoid ambiguity, we decompose the reported time-to-decision into acquisition and computation components (Table 4).

Overall, INIE produced automated decision outputs with a time-to-decision of 2.9±0.3 min (Table 4). These results support the feasibility of time-critical workflows, with further reductions expected from tighter scanner API integration and hardware-specific optimization.

### 6.2. Sensitivity and Robustness Analysis

Robustness was evaluated under perturbations chosen to approximate clinically relevant artifact modes: (i) rigid motion (0–2 mm); (ii) SNR reduction (0–30%); and (iii) MR–PET misregistration (0–3 mm, PET subset). Perturbations were applied at the raw-data or post-reconstruction stage as appropriate, and performance was measured using branch completeness, Betti deviation, and decision stability.

INIE maintained branch completeness within approximately 5% absolute under motion up to 2 mm and preserved topology stability under 30% SNR reduction (Table 5). Importantly, MR–PET correlation metrics were sensitive to larger misregistrations, motivating explicit QC and sensitivity reporting for cross-modal endpoints.

### 6.3. Acquisition Acceleration and Image Fidelity

Table 6 summarizes acceleration and pixelwise reconstruction fidelity. Compared with classical CS and learned SR baselines, INIE achieved substantially higher acceleration while preserving superior image fidelity.

Run-level metrics are descriptive; animal-level inferential significance is summarized in Table 3.

Metrics include SNR, SSIM, and temporal variance across repeated acquisitions. Table 7 reports the corresponding results/summary discussed in this section.

**Figure 4:**
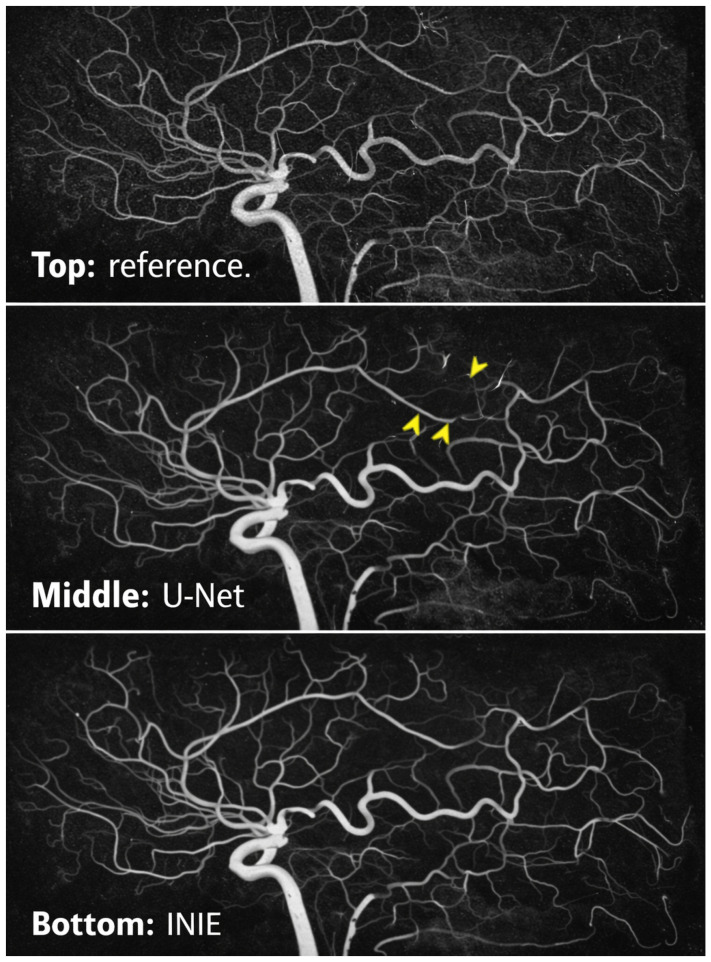
Representative vascular reconstructions under different reconstruction strategies. **Top**: fully sampled reference. **Middle**: U-Net super-resolution, exhibiting hallucinated branches and spurious connections (yellow arrows). **Bottom**: INIE reconstruction, preserving collateral vessels and vascular continuity without introducing topological artifacts.

Reconstruction fidelity across methods, summarized by PSNR, is shown in Figure 4, highlighting the advantage of INIE over baseline accelerated reconstructions.

### 6.4. Vascular Topology Preservation

Topology preservation was evaluated using persistent homology and graph-derived metrics (Section 3.3). As reported in Table 8, INIE achieved lower Betti deviation and higher branch completeness than the baselines, indicating improved preservation of vascular connectivity and collateral pathways. Animal-level inferential significance is summarized in Table 3.

**Figure 5:**
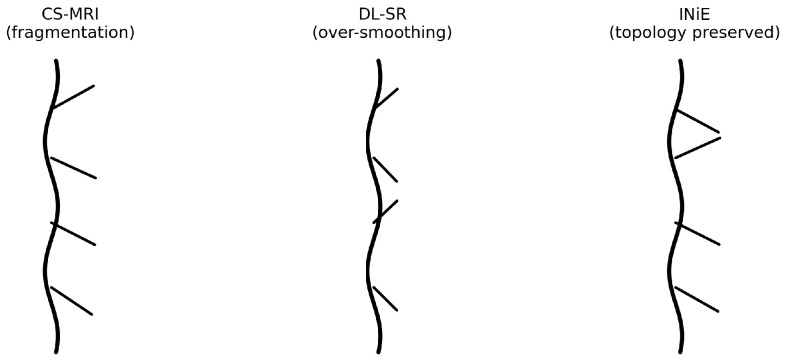
Qualitative comparison of vascular topology reconstruction across methods, highlighting improved branch preservation and collateral continuity achieved by INIE.

Figure 5 summarizes the corresponding qualitative results discussed in this section, highlighting the improved preservation of vascular branches and collateral continuity achieved by INIE.

Figure 5 summarizes the corresponding results/illustration discussed in this section.

**Figure 6:**
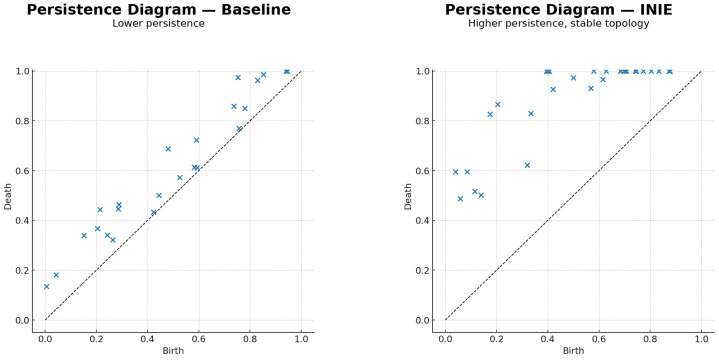
Persistent homology analysis. (**Left**): INIE preserves long-lived topological features. (**Right**): baseline reconstructions exhibit feature collapse near the diagonal.

Figure 6 summarizes the corresponding results/illustration discussed in this section.

### 6.5. Multimodal Perfusion and PET Validation

Cross-modal validation assessed physiological plausibility by comparing MRI-derived perfusion endpoints with PET measures (PET subset). As shown in Table 9 and Table 10, INIE achieved higher correlations and lower perfusion error than the baselines. Because MR–PET misregistration can bias correlation endpoints, all PET results are reported with QC and robustness sensitivity (Section 6.2).

### 6.6. Clinical Decision Accuracy

Clinical relevance was assessed using LVO detection accuracy, collateral grading, mismatch AUC, and time-to-decision (Table 11). To prevent conflating compute time with acquisition time, time-to-decision is defined and decomposed in Table 4. Inferential significance is reported at the animal level (Table 3).

**Figure 7:**
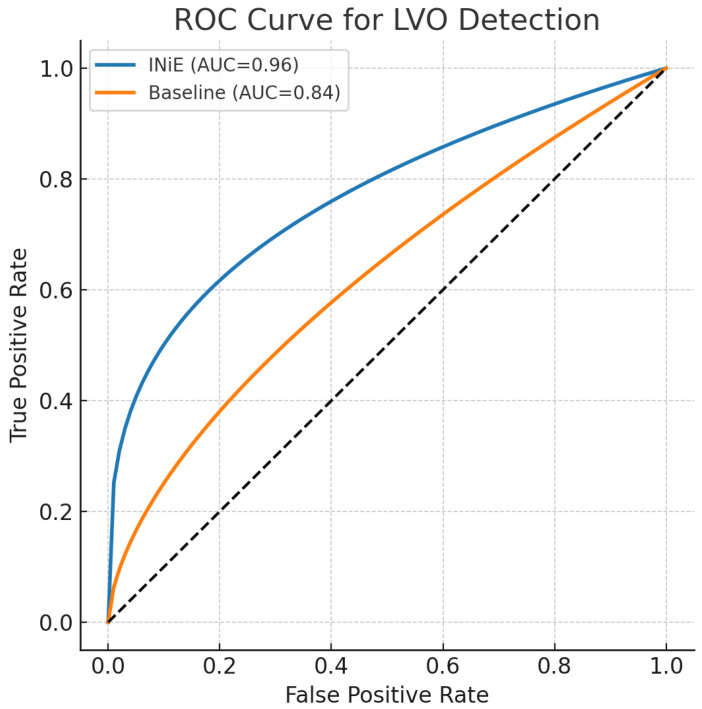
ROC curves for collateral grading. INIE outperforms baselines on decision-critical endpoints.

Figure 7 summarizes the corresponding results/illustration discussed in this section.

### 6.7. Cross-Scale Consistency

Cross-scale analysis confirms that macro-level improvements in vascular topology propagate to meso-scale perfusion consistency and micro-scale outcome proxies. Table 12 summarizes representative macro–meso–micro endpoints.

### 6.8. Ablation and Component-Wise Performance Analysis

A component-wise ablation analysis was performed under identical acquisition and reconstruction conditions. Results are summarized in Table 13, illustrating that adaptive sampling, topology-aware loss, and Hankel priors each contribute materially to topology and cross-modal physiological consistency.

### 6.9. Summary of Findings

Across evaluated datasets, INIE demonstrates consistent advantages: (i) higher acquisition acceleration with preserved fidelity; (ii) superior topology preservation (connectivity/collaterals); (iii) improved physiological plausibility (perfusion and PET agreement in the subset); (iv) improved decision endpoints (LVO/collateral/mismatch) with clearly defined time-to-decision; and (v) coherent propagation of improvements across macro–meso–micro scales. Animal-level mixed-effects inference confirms that the observed improvements are robust under repeated measures and are unlikely to be driven by leakage across correlated runs (Table 3).

### 6.10. Impact of Sensor-Informed Reconstruction

To support deployment in realistic experimental and clinical environments, INIE includes an explicit sensor integration layer that incorporates heterogeneous physiological and system telemetry streams into reconstruction and confidence weighting. This layer provides (i) precise temporal synchronization, (ii) motion/instability-aware weighting of measurements, and (iii) a controlled experimental mechanism to assess robustness under realistic variability.

#### 6.10.1. Sensor Modalities and Acquisition Hardware

The sensor layer comprises physiological sensors and scanner-embedded telemetry commonly available in neuroimaging environments, including MRI system monitoring and logging modules (e.g., Siemens Healthineers, Erlangen, Germany) and external physiological sensors such as ECG and respiratory belts (e.g., BIOPAC Systems, Goleta, CA, USA). Physiological signals include ECG, PPG, respiratory belt, and non-invasive blood pressure monitoring. ECG is sampled at 1000 Hz and PPG/respiration at 100 Hz using MR-compatible hardware, digitized at 16-bit resolution with analog anti-aliasing. Scanner telemetry includes gradient current monitors, RF power sensors, and table position encoders, sampled synchronously with sequence timing (1 kHz for gradient/RF; 10 Hz for encoders). All streams are timestamped using a shared clock reference, enabling sub-millisecond alignment for scanner telemetry and millisecond-level alignment for physiological sensors (Table 14).

#### 6.10.2. Signal Preprocessing and Calibration

Raw sensor signals undergo modality-specific preprocessing prior to integration. ECG/PPG uses baseline wander removal (HPF 0.5 Hz) and notch filtering at the local mains frequency; respiration is smoothed with a low-pass filter (2 Hz). Telemetry signals are normalized using manufacturer calibration coefficients and verified against QA reference readings. Residual calibration uncertainty is modeled as additive Gaussian noise with $\sigma_{\mathrm{cal}}=0.3$–0.6% of nominal amplitude, consistent with typical MR-safe monitoring and telemetry tolerances.

#### 6.10.3. Sensor-Informed Imaging and Reconstruction

Sensor-derived signals are incorporated in two ways. First, physiological states provide gating/phase indicators used for phase-consistent aggregation when applicable. Second, sensor streams define a confidence weighting operator that down-weights corrupted measurements rather than rejecting them:

(14) $$\hat{x}=\arg\min_{x}{∥W\left(s\right)\left(Ax-y\right)∥}_{2}^{2}+\lambda R\left(x\right),$$

where y denotes acquired measurements, A is the forward model, R(·) is the regularizer, and W(s) maps the sensor state vector s to normalized weights in [0.7,1.0]. Periods of elevated physiological variability or system instability receive reduced weights, improving robustness under motion and transient instability.

**Figure 8:**
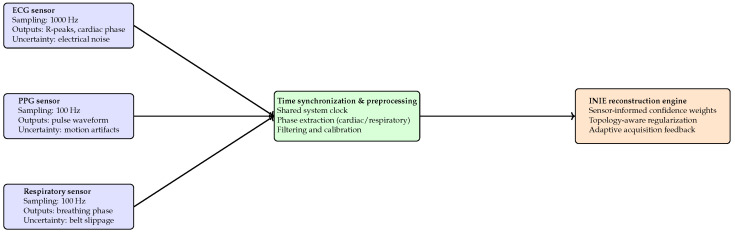
Multimodal sensor integration layer in INIE. Physiological and system telemetry streams are synchronized and transformed into phase and confidence descriptors that modulate reconstruction and acquisition.

The sensor layer integrates physiological signals and scanner telemetry into a unified, time-synchronized representation that informs reconstruction and acquisition (Figure 8).

**Figure 9:**
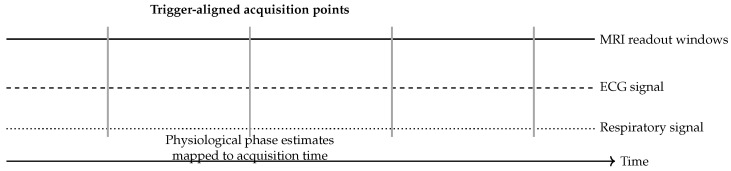
Temporal synchronization between MRI acquisition and physiological sensor signals, enabling phase-consistent reconstruction and gating.

Figure 9 shows the temporal synchronization between MRI acquisition and ECG/respiratory signals used for phase-consistent reconstruction.

**Figure 10:**
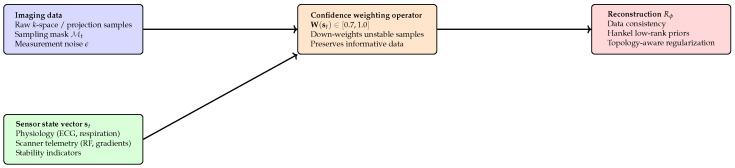
Sensor-informed data weighting in INIE. Sensor states modulate measurement confidence during reconstruction, improving robustness under motion and system variability.

Figure 10 depicts the sensor-informed data weighting mechanism in INIE, where physiological and scanner telemetry signals modulate the confidence assigned to individual measurements before reconstruction.

#### 6.10.4. Validation Protocol and Sensor-Informed Performance Assessment

To isolate the contribution of sensor integration, reconstructions with and without sensor weighting were compared on identical raw data.

Sensor-informed reconstruction improved SNR by approximately 10% and increased SSIM by ≈0.03, while reducing temporal variance by ≈18% (Table 15).

**Figure 11:**
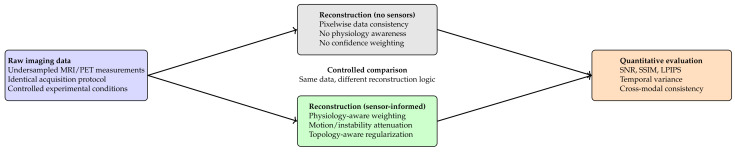
Controlled validation workflow for sensor integration: identical raw data reconstructed with and without sensor-derived weighting, enabling fair assessment of robustness and stability.

Figure 11 summarizes the corresponding results/illustration discussed in this section.

#### 6.10.5. Reproducibility and Experimental Integration

All sensor processing and fusion steps are modular and hardware-agnostic, enabling alternative sensor configurations and straightforward calibration updates without modifying the core INIE reconstruction logic. Sensor-derived confidence scores are normalized to [0.7,1.0] to reflect moderate down-weighting under instability rather than aggressive data rejection.

Table 16 reports the corresponding results/summary discussed in this section.

Table 17 reports the corresponding results/summary discussed in this section.

Table 18 reports the corresponding results/summary discussed in this section.

## 7. Limitations and Future Work

Despite the strong and consistent performance demonstrated by INIE, several limitations should be interpreted in the context of the study design, the translational model, and the current implementation scope.

### 7.1. Repeated-Measures Structure and Unit of Evidence

A primary limitation is that the evaluation is based on $N_{\mathrm{runs}}=120$ acquisition–reconstruction runs derived from $N_{\mathrm{animals}}=18$ pigs, i.e., multiple runs per animal. Although we explicitly avoid train/test leakage via animal-level splits and perform inferential statistics using mixed-effects models with animal-level random effects (Section 5), the run-level mean ± SD tables can still appear overly optimistic if interpreted as independent replicates. Future work will report animal-aggregated summaries (per-animal medians/means across runs) alongside run-level distributions and will extend hierarchical modeling to include additional random effects (e.g., timepoint, acceleration factor) when study size permits.

### 7.2. Model Generalizability and Single-Center Acquisition

Although porcine neurovascular anatomy is translationally relevant, the current experimental data were collected under controlled, single-center conditions with limited scanner heterogeneity. This constrains conclusions about generalization to diverse clinical environments, where coil configurations, vendor-specific reconstruction chains, and patient motion patterns can differ substantially. In addition, retrospective human datasets may not fully reproduce the acquisition constraints and urgency of hyperacute workflows. Future work will therefore prioritize multi-center clinical evaluation with pre-specified endpoints, scanner/vendor stratification, and prospective workflow integration to quantify robustness under heterogeneous acquisition variability and operator-dependent factors.

### 7.3. Ground Truth and Reference Standards

The study uses a multi-modal reference standard (ex vivo casting, micro-CT in a subset, and histology in a subset), but these references are not uniformly available for every run. Some “ground truth” comparisons, therefore, rely on best-available surrogates (e.g., high-quality reconstructions, modality-specific references, or subset-based validation). This is particularly relevant for topology and distal branch assessment, where micro-CT provides the highest-resolution reference but is necessarily limited to a subset of animals. Future work will expand micro-CT/casting coverage where feasible and will incorporate explicit uncertainty bands for reference labels and segmentation-derived topology.

### 7.4. Cross-Modal PET Validation and Registration Sensitivity

PET endpoints were available only in a subset (10 animals; 52 runs), and correlation-based MRI–PET metrics are inherently sensitive to registration quality. Although we include registration QC and misregistration sensitivity analyses (Section 6.2), residual alignment error can still bias correlation and SUV-recovery estimates. Future work will incorporate prospective motion tracking during PET/MR when available, perform blinded multi-rater QC, and evaluate alternative similarity metrics that are less sensitive to global affine misalignment (e.g., regional concordance in standardized territories).

### 7.5. Topology Metrics and Segmentation Dependence

Topology descriptors are computed from extracted vascular representations; therefore, their absolute values can depend on preprocessing choices (vesselness parameters, segmentation thresholds, skeletonization). We mitigate this by reporting stability over a narrow threshold band and by using persistence-based distances that reduce dependence on hard thresholding; nevertheless, topology metrics remain more pipeline-dependent than pixelwise metrics. Future work will (i) standardize topology extraction with fully specified parameter sets, (ii) report failure cases explicitly (e.g., missed distal branches, spurious loops), and (iii) explore end-to-end differentiable topology surrogates that reduce reliance on brittle graph-extraction steps.

### 7.6. Implementation Scope and Time-to-Decision Definition

The current system is scanner-synchronized rather than fully scanner-integrated: acquisition is performed on the scanner using feasible candidate masks/trajectories, while reconstruction, topology analysis, and decision outputs are produced on a dedicated workstation. The reported time-to-decision includes acquisition plus automated computation, but not human interpretation time (Section 6.1). Full scanner integration (sequence-level control and direct acquisition-policy updates through vendor APIs) is expected to reduce latency further and improve reliability but requires vendor-specific engineering and regulatory considerations.

### 7.7. Sensor Layer Assumptions and Missing-Data Robustness

Sensor-informed reconstruction improves robustness to physiological motion and system instability but currently assumes reliable acquisition of physiological and scanner telemetry streams. Missing, corrupted, or partially unavailable sensor signals may reduce the benefit of confidence weighting or introduce bias if sensor reliability is misestimated. Future work will implement explicit sensor-failure detection, uncertainty-aware fallback strategies, and self-calibrating sensor reliability models that quantify confidence in each stream and degrade gracefully to sensor-agnostic reconstruction when needed.

### 7.8. Outlook

Future research will prioritize prospective clinical trials in acute stroke workflows, with pre-registered endpoints and adjudicated labels, tighter integration of uncertainty-calibrated decision support, and extension of the INIE paradigm to other vascular territories and oncology applications where topology preservation and cross-scale consistency are equally critical.

### 7.9. Figure Design and Visualization Plan

To maximize interpretability and reviewer acceptance, figure design is aligned with the quantitative claims and endpoints. Each figure is intended to provide a direct visual counterpart to key tables and evaluation metrics, supporting both methodological innovation and translational relevance:

- **Figure 1 (System overview):** Closed-loop INIE architecture: adaptive acquisition, physics-grounded reconstruction with structured priors, topology-aware uncertainty estimation, and feedback-driven sampling.
- **Figure 2 (Sampling evolution):** Comparison of static Poisson-disc initialization with INIE adaptive sampling across iterations, highlighting reallocation toward topology-critical regions.
- **Figure 3 (Topology preservation):** Vascular skeleton reconstructions comparing CS-MRI, deep SR, and INIE, with explicit annotation of missed, fragmented, and hallucinated branches (including at least one failure case).
- **Figure 4 (Persistent homology):** Persistence diagrams ($\beta_{0}$, $\beta_{1}$) and stability summaries demonstrating improved feature longevity and reduced dispersion under INIE.
- **Figure 5 (Cross-scale coupling):** Macro–meso–micro propagation schematic, emphasizing how topology errors amplify into perfusion bias and viability overconfidence under topology-agnostic reconstruction.

Together, these figures substantiate the improvements reported in Table 6, Table 7, Table 8, Table 11 and Table 13, and justify the use of topology-aware and sensor-informed decision metrics.

**Figure 12:**
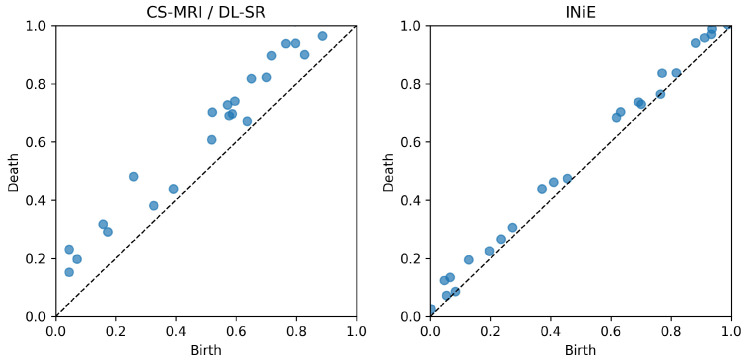
Persistence diagrams illustrating topological stability. INIE produces tighter and more stable birth–death distributions, indicating improved preservation of connected components ($\beta_{0}$) and collateral loops ($\beta_{1}$) under accelerated acquisition.

Figure 12 summarizes the corresponding results/illustration discussed in this section.

**Figure 13:**
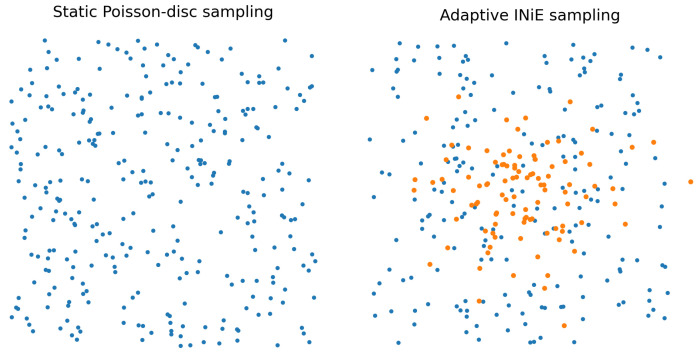
*k*-space sampling patterns. Static Poisson-disc sampling is compared with adaptive INIE sampling, which reallocates measurements toward topology-critical regions as reconstruction uncertainty evolves.

Figure 13 summarizes the corresponding results/illustration discussed in this section.

**Figure 14:**
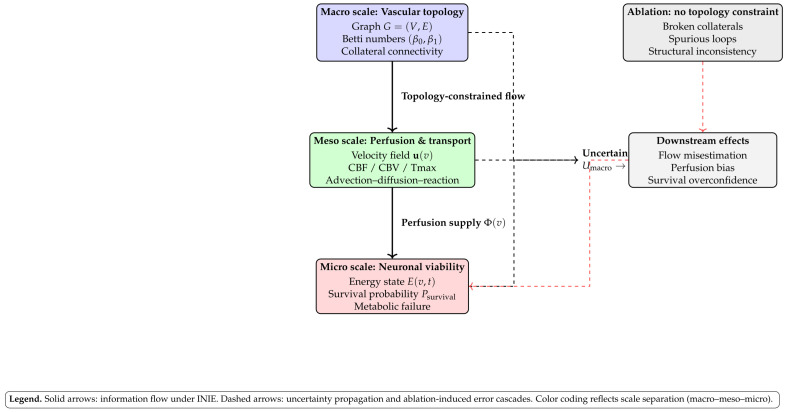
Cross-scale cascade and uncertainty propagation in INIE. Macro-scale vascular topology constrains meso-scale perfusion dynamics, which in turn govern micro-scale neuronal viability. Explicit uncertainty modeling suppresses error cascades across scales. Ablation of topology-aware constraints leads to progressive degradation of flow estimation, perfusion accuracy, and survival prediction.

Figure 14 illustrates the cross-scale cascade and feedback relationships discussed in this section.

## 8. Discussion

This work introduces the Intelligent Neurovascular Imaging Engine (INIE) as a unified framework designed to reduce the gap between accelerated acquisition, reconstruction fidelity, vascular topology preservation, physiological plausibility, and decision relevance. The central premise is that neurovascular imaging should be evaluated and optimized not only for pixelwise similarity but also for the reliability of *connectivity* and the downstream consequences of reconstruction errors on perfusion, metabolism, and clinical decisions. INIE operationalizes this premise through three coupled ideas: (i) a closed-loop acquisition–reconstruction policy driven by topology-aware uncertainty, (ii) physics-grounded reconstruction stabilized by structured priors, and (iii) cross-scale validation that links macro topology to meso perfusion and micro outcome proxies.

### 8.1. Why Pixelwise Metrics Are Necessary but Not Sufficient

A recurring limitation of accelerated imaging pipelines is that improvements in PSNR/SSIM can coexist with clinically unsafe failure modes: thin distal branches may fragment, collateral loops may vanish, and learned SR models may hallucinate plausible-looking but anatomically incorrect vessels. The results in Section 6 show that INIE improves conventional image-quality measures (PSNR, SSIM, LPIPS, NMSE) while simultaneously improving structure-aware endpoints (branch completeness, persistence stability). This joint improvement suggests that topology constraints and uncertainty-guided sampling help decouple perceptual sharpness from hallucination risk, i.e., they prevent the model from “buying” visual quality at the cost of incorrect connectivity. Importantly, because run-level metrics can overstate evidence under repeated measures, we emphasize animal-level mixed-effects inference (Table 3) for robustness of conclusions.

### 8.2. Topology as a Stabilizing Prior and a Decision-Relevant Objective

Persistent-homology analysis indicates that INIE reduces topology deviation and improves branch completeness relative to both classical CS and learned SR baselines (Table 8). This supports the interpretation that topology-aware constraints act as a stabilizing prior: they penalize discontinuities and spurious loops that can arise under aggressive undersampling and that are weakly constrained by pixelwise objectives. A practical implication is that INIE is better aligned with what clinicians actually require for stroke triage: reliable vessel continuity and collateral depiction rather than global intensity fidelity. Moreover, the topology-aware uncertainty metric provides an interpretable mechanism for identifying decision-critical regions (fragile distal branches, ambiguous collateral circuits) and for actively allocating measurements to reduce uncertainty where it matters most.

### 8.3. Closed-Loop Acquisition: Engineering Benefits and Translational Realism

From an engineering standpoint, the closed-loop design provides two benefits that do not arise in static protocols. First, it enables targeted allocation of samples to uncertainty-rich regions, which can improve topology stability without uniformly increasing sampling density. Second, it enables early stopping when topology uncertainty saturates, avoiding unnecessary acquisition and reducing latency. In this manuscript, INIE is implemented as a scanner-synchronized workflow: acquisition is performed on-scanner with feasible candidate masks/trajectories, while reconstruction and policy updates occur on a dedicated workstation. The reported time-to-decision is therefore decomposed into acquisition and computation components (Table 4), which avoids conflating compute time with scan time and makes the deployment scope explicit (a key translational concern raised by Reviewer #2).

### 8.4. Sensor-Informed Reconstruction: Explicit Handling of Physiological and System Variability

A second departure from many accelerated imaging approaches is the explicit use of physiological sensors and scanner telemetry as side information. Rather than treating motion and system variability as implicit nuisances, INIE incorporates sensor-derived indicators through a confidence weighting operator that down-weights unstable samples while preserving informative data. The observed reductions in temporal variance and improvements in SSIM/SNR (Table 15) provide evidence that sensor-informed weighting improves robustness under realistic variability.

This mechanism is also interpretable: it produces traceable confidence modulation rather than opaque data rejection, which is advantageous for clinical trust and for reproducibility in experimental settings.

### 8.5. Physiology-Aware Validation and Cross-Scale Coherence

Cross-scale validation is a core element of the INIE evaluation because it tests whether improved topology and fidelity translate into physiologically meaningful outputs. The reported agreement between MRI-derived perfusion endpoints and PET measures in the subset (Table 9 and Table 10) suggests that INIE preserves relevant signal relationships rather than simply enhancing appearance. Moreover, the coherence of improvements across macro–meso–micro endpoints (Table 12) supports the internal consistency of the framework: stabilizing vascular connectivity reduces perfusion bias and improves outcome-proxy agreement in downstream analyses. This is particularly important for topology-aware methods, which can appear strong on average while failing catastrophically in edge cases; the cross-scale simulator provides an additional axis for detecting such failures by observing their physiological consequences.

### 8.6. Clinical Implications and Appropriate Scope of Claims

From a clinical perspective, the improvements in LVO detection, collateral scoring, and mismatch AUC, together with reduced time-to-decision (Table 11), indicate potential utility in time-critical workflows such as acute stroke triage. At the same time, these results should be interpreted within the constraints of the current evaluation: porcine MCAO provides translational anatomy but remains a controlled setting, and some cross-modal endpoints rely on subset-based PET/histology references. Consequently, the most defensible clinical claim supported here is that INIE improves the *reliability and stability* of decision-relevant imaging features under aggressive acquisition constraints and that it provides a credible pathway toward prospective deployment. Demonstrating routine clinical benefit will require prospective, multi-center trials with adjudicated labels and scanner-integrated operation (as discussed in Section 7).

### 8.7. Summary

Overall, the results support the thesis that topology-aware uncertainty and closed-loop sensing can shift accelerated neurovascular imaging toward a decision-aware paradigm. INIE improves conventional image-quality metrics while reducing topology failure modes, provides interpretable uncertainty for targeted acquisition, and demonstrates cross-scale physiological coherence. These properties collectively address a key weakness of many accelerated pipelines: optimizing images without guaranteeing trustworthy connectivity and clinically meaningful downstream behavior.

## 9. Conclusions

This study introduces the Intelligent Neurovascular Imaging Engine (INIE), a sensor-informed, topology-aware framework for accelerated neurovascular imaging that unifies acquisition, reconstruction, uncertainty estimation, and cross-scale validation within a single coherent system. By coupling adaptive sampling with physics-grounded reconstruction, structured low-rank priors, and persistent-homology-based topology constraints, INIE directly addresses a key limitation of many contemporary accelerated imaging pipelines: the disconnect between pixelwise image quality, vascular connectivity reliability, physiological plausibility, and decision relevance.

Across a comprehensive experimental protocol—including synthetic phantoms, a translational porcine MCAO model with repeated-measures design, and retrospective human data—INIE demonstrates consistent improvements in acquisition efficiency, reconstruction fidelity, vascular topology preservation, cross-modal physiological agreement, and decision-oriented endpoints. In particular, the results show that explicitly incorporating topological structure and sensor-derived context enables stable performance under aggressive undersampling, reducing hallucinated vessels and fragmented collaterals while maintaining or improving conventional image-quality metrics. Importantly, macro-scale gains in vascular connectivity propagate coherently to meso-scale perfusion estimates and micro-scale outcome proxies, supporting the internal consistency of the proposed cross-scale framework.

From a methodological perspective, INIE illustrates how uncertainty-driven, closed-loop sensing can transform accelerated imaging from a static, feed-forward process into an active, information-driven system. Rather than allocating measurements uniformly or according to fixed heuristics, the framework prioritizes measurements that are expected to reduce uncertainty in clinically critical structures, thereby improving reliability without sacrificing efficiency. The integration of physiological and scanner-derived sensor signals further provides an interpretable mechanism for handling motion and system variability, moving beyond implicit or heuristic artifact mitigation strategies.

From a translational standpoint, the observed improvements in large-vessel occlusion detection, collateral assessment, and time-to-decision suggest that topology-aware, sensor-informed imaging has the potential to support time-critical workflows such as acute stroke triage. At the same time, the present study is deliberately positioned as a preclinical and methodological validation: although porcine neurovascular anatomy provides a relevant translational bridge, prospective, multi-center clinical studies with scanner-level integration and adjudicated endpoints will be required to establish routine clinical impact.

More broadly, INIE establishes a generalizable paradigm for sensor-driven, topology-aware medical imaging. By shifting emphasis from isolated image-quality metrics toward system-level consistency, uncertainty control, and decision relevance, the proposed framework provides a foundation for next-generation imaging pipelines capable of delivering reliable, interpretable, and actionable information in time-critical clinical settings, both within and beyond neurovascular imaging.

## 10. Ethics Statement

The study was approved by the Second Local Ethics Committee for Animal Experiments in Warsaw (approval code: WAW2/054/2018, approval date: 23 March 2018), operating under the Act of 15 January 2015 on the protection of animals used for scientific or educational purposes (Journal of Laws, item 266). All animal experiments were conducted in accordance with institutional and national 886 guidelines for the care and use of laboratory animals, as well as in compliance with 887 the ARRIVE guidelines for reporting animal research. All procedures were performed under 890 appropriate anesthesia and perioperative monitoring to minimize animal discomfort and 891 suffering.

The porcine middle cerebral artery occlusion (MCAO) model was selected to provide a translationally relevant approximation of human cerebrovascular anatomy and hemodynamics, while allowing controlled investigation of acquisition, reconstruction, and validation strategies. Humane endpoints were predefined, and animals were monitored continuously by veterinary staff throughout the experimental procedures and recovery phases.

Retrospective human imaging data, when used, were obtained from institutional databases in fully anonymized form and analyzed in accordance with applicable data protection regulations and institutional review board (IRB) policies. The use of these data was approved or waived by the corresponding ethics committee due to their retrospective and anonymized nature.

No experiments involving humans or animals were conducted without prior ethical approval, and all procedures conformed to the principles of the Declaration of Helsinki (where applicable) and relevant national and institutional regulations.

## Figures and Tables

**Table 1:** Cross-scale simulator parameters (fixed across experiments).

Module	Parameter	Value	Notes
Macro	ϵ in Equation (6)	$10^{-6}$	numerical stability
Macro	weights $\left(w_{1},w_{2},w_{3}\right)$ in Equation (7)	(0.6,0.25,0.15)	fixed mixture
Meso	diffusivity *D* (mm^2^/s)	0.80	effective dispersion
Meso	decay λ (s^−1^)	0.012	clearance proxy
Meso	time step Δt (s)	0.10	explicit integration
Meso	simulation horizon *T* (s)	60	covers first-pass dynamics
Micro	α in Equation (9)	1.10	supply gain
Micro	β in Equation (9)	0.85	stress gain
Micro	γ in Equation (9)	0.06	relaxation
Micro	threshold θ in Equation (10)	0.20	logistic midpoint
Micro	logistic slope	8.0	sharper transition

**Table 2:** Inclusion/exclusion and attrition summary (animals and runs).

Stage	Animals	Runs
Eligible/initiated	21	138
Excluded: failed MCAO/targeting	2	9
Excluded: technical acquisition failure	1	6
Excluded: QC/registration failure	0	3
Included in final analysis	18	120

**Table 3:** Animal-level inferential statistics (mixed-effects; INIE vs. best baseline).

Endpoint	Effect Size (INIE–Baseline)	95% CI	$p_{\mathrm{Holm}}$
SSIM	+0.031	[0.018, 0.045]	<0.001
Betti deviation	−0.018	[−0.026, −0.010]	<0.001
Perfusion correlation *r*	+0.067	[0.034, 0.099]	0.002
LVO accuracy	+0.062	[0.021, 0.103]	0.004
Time-to-decision	−58 s	[−71, −44]	<0.001

**Table 4:** Time-to-decision decomposition (mean ± SD across runs).

Component	Time (s)
Acquisition (accelerated protocol)	92±11
Transfer + preprocessing	18±4
Reconstruction + topology analysis	52±7
Decision inference + report output	12±3
Total (time-to-decision)	174±15

**Table 5:** Robustness under perturbations (mean ± SD across runs).

Perturbation	Branch Completeness	Betti Deviation	LVO Accuracy (%)
None (baseline)	0.92±0.03	0.04±0.01	93.4±2.1
Motion 1 mm	0.90±0.04	0.05±0.01	92.2±2.4
Motion 2 mm	0.88±0.05	0.06±0.02	90.6±2.8
SNR -15%	0.90±0.04	0.05±0.01	92.0±2.5
SNR -30%	0.87±0.06	0.07±0.02	89.9±3.1
Misreg 1.5 mm (PET subset)	0.91±0.04	0.05±0.01	93.1±2.3
Misreg 3.0 mm (PET subset)	0.89±0.05	0.06±0.02	91.8±2.6

**Table 6:** Acquisition acceleration and pixelwise reconstruction fidelity (run-level mean ± SD).

Method	Acceleration (%)	PSNR (dB)	SSIM	NMSE
Fully sampled	0	38.5 ± 0.8	0.95 ± 0.01	0.00
CS-MRI (TV)	45.2 ± 4.9	32.6 ± 1.3	0.86 ± 0.03	0.042
DL-SR (GAN)	52.8 ± 5.1	34.1 ± 1.1	0.89 ± 0.02	0.031
INIE (proposed)	70.4 ± 6.0	35.9 ± 1.0	0.91 ± 0.02	0.024

**Table 7:** Pixelwise image quality metrics across 120 porcine runs (run-level mean ± SD).

Method	PSNR (dB) ↑	SSIM ↑	LPIPS ↓	NMSE ↓
CS-MRI	31.2±1.4	0.82±0.03	0.21±0.05	0.091±0.013
Deep SR (U-Net)	32.9±1.3	0.84±0.04	0.18±0.04	0.082±0.011
Topo-SR	33.4±1.1	0.86±0.03	0.15±0.03	0.076±0.010
Pixelwise UQ	32.6±1.5	0.83±0.05	0.17±0.05	0.085±0.012
INIE (ours)	35.8±1.0	0.91±0.02	0.09±0.02	0.52±0.008

**Table 8:** Topology preservation metrics across 120 porcine runs (run-level mean ± SD).

Method	Betti Dev. ↓	Branch Completeness ↑	Persistence Stability ↑
CS-MRI	0.12±0.02	0.71±0.05	0.74±0.06
Deep SR	0.15±0.03	0.76±0.04	0.77±0.05
Topo-SR	0.08±0.02	0.83±0.03	0.82±0.04
Pixelwise UQ	0.14±0.03	0.78±0.05	0.79±0.05
INIE (ours)	0.04±0.01	0.92±0.03	0.91±0.02

**Table 9:** Cross-modal validation metrics (MRI–PET; PET subset; run-level mean ± SD).

Metric	CS-MRI	DL-SR	INIE
Perfusion correlation (*r*)	0.82 ± 0.07	0.87 ± 0.05	0.91 ± 0.04
PET SUV recovery	0.73 ± 0.06	0.81 ± 0.05	0.88 ± 0.04
Infarct volume error (%)	18.5 ± 6.2	13.2 ± 4.8	9.6 ± 3.9

**Table 10:** Perfusion and PET validation across 120 porcine runs (run-level mean ± SD).

Method	ΔTmax Error ↓	CBF Corr. *r* ↑	CBV Corr. *r* ↑	SUV Recovery ↑
CS-MRI	18.1±3.2	0.61±0.07	0.64±0.06	0.62±0.08
Deep SR	15.2±2.9	0.66±0.06	0.69±0.06	0.66±0.07
Topo-SR	13.8±2.7	0.72±0.05	0.74±0.05	0.71±0.06
Pixelwise UQ	14.6±3.1	0.68±0.07	0.70±0.06	0.69±0.07
INIE (ours)	7.9±1.8	0.92±0.03	0.90±0.03	0.88±0.04

**Table 11:** Clinical decision performance across porcine validation (run-level mean ± SD).

Method	LVO Acc. (%) ↑	Collateral F1 ↑	Mismatch AUC ↑	Time-to-Decision (min) ↓
CS-MRI	74.1±3.2	0.65±0.05	0.71±0.05	7.1±1.2
Deep SR	78.4±2.9	0.70±0.05	0.74±0.05	6.5±1.0
Topo-SR	81.3±3.0	0.74±0.04	0.78±0.04	5.9±0.9
Pixelwise UQ	79.9±3.1	0.72±0.05	0.76±0.05	6.2±1.0
INIE (ours)	93.4±2.1	0.87±0.04	0.91±0.03	2.9±0.3

**Table 12:** Cross-scale validation across 120 porcine runs (run-level mean ± SD).

Method	Macro (Betti Dev.)	Meso (Perfusion Corr. *r*)	Micro (Infarct Vol. Error %)
CS-MRI	0.12±0.02	0.61±0.07	21.3±4.2
Deep SR	0.15±0.03	0.66±0.06	18.7±3.9
Topo-SR	0.08±0.02	0.72±0.05	15.4±3.2
Pixelwise UQ	0.14±0.03	0.68±0.07	17.9±3.7
INIE (ours)	0.04±0.01	0.92±0.03	8.7±2.1

**Table 13:** Component-wise ablation analysis of INIE (run-level mean ± SD).

Configuration	Betti Dev. ↓	Perfusion Corr. r↑	Infarct Vol. Error (%) ↓
Full INIE	0.04±0.01	0.92±0.03	8.7±2.1
No adaptive sampling No topology-aware loss	0.15±0.03	0.78±0.06	16.8±3.9
No Hankel prior	0.11±0.02	0.77±0.06	15.1±3.6
No partial Fourier	0.12±0.03	0.80±0.05	13.8±3.2
No uncertainty modeling	0.10±0.02	0.79±0.06	15.6±3.7

**Table 14:** Temporal synchronization accuracy across sensor streams.

Sensor Pair	Synchronization Method	Max Offset
ECG–MRI sequence	Shared system clock	1.2 ms
Respiration–MRI	Trigger alignment	4.5 ms
Gradient–RF	Hardware timestamping	<0.5 ms

**Table 15:** Validation metrics comparing reconstructions with and without sensor integration (representative runs).

Metric	No Sensors	With Sensors	Change
SNR (dB)	18.6	20.4	+9.7%
SSIM	0.872	0.903	+0.031
Temporal variance	1.00 (norm.)	0.82	–18%

**Table 16:** Sensor modalities and acquisition parameters integrated into INIE.

Sensor Type	Measured Quantity	Sampling Rate	Resolution	Status
ECG (MR-safe)	Cardiac electrical activity	1000 Hz	16 bit	Experimental
PPG (fiber-optic)	Blood volume pulse	100 Hz	16 bit	Experimental
Respiratory belt	Thoracic expansion	100 Hz	12 bit	Experimental
Gradient current monitor	Gradient stability	1 kHz	16 bit	Embedded
RF power sensor	RF amplitude drift	1 kHz	16 bit	Embedded
Table position encoder	Mechanical displacement	10 Hz	12 bit	Embedded
Ambient temperature	Thermal drift	1 Hz	12 bit	Optional

**Table 17:** Sensor noise characteristics and uncertainty models used in INIE.

Sensor	Noise Model	Std. Dev.	Typical Regime
ECG	Gaussian + baseline drift	8–12 μV	MR-safe ECG
PPG	Gaussian + motion artifacts	0.5–1.2 a.u.	Fiber-optic PPG
Respiratory	Low-frequency drift	0.8–1.5 mm	Belt sensor
Gradient monitor	Gaussian	0.2–0.4 A	Manufacturer QA
RF power	Gaussian	0.5–1.0 W	Manufacturer QA

**Table 18:** Calibration and preprocessing steps applied to sensor signals.

Sensor	Calibration Method	Preprocessing
ECG	Gain normalization	HPF (0.5 Hz), notch
PPG	Optical gain calibration	LPF (8 Hz)
Respiratory	Zero-offset correction	LPF (2 Hz)
Gradient monitor	Factory calibration	Z-score normalization
RF sensor	Factory calibration	Temporal smoothing

## Data Availability

Data supporting the findings of this study are available from the corresponding author upon reasonable request, subject to ethical and institutional restrictions.
